# 2′-Fucosyllactose Is Well Tolerated in a 100% Whey, Partially Hydrolyzed Infant Formula With *Bifidobacterium lactis*: A Randomized Controlled Trial

**DOI:** 10.1177/2333794X19833995

**Published:** 2019-03-15

**Authors:** Heidi M. Storm, Julie Shepard, Laura M. Czerkies, Brian Kineman, Sarah S. Cohen, Heidi Reichert, Ryan Carvalho

**Affiliations:** 1 Nestlé Nutrition, Florham Park, NJ, USA; 2 Ohio Pediatric Research Association, Inc, Dayton, OH, USA; 3 EpidStat Institute, Ann Arbor, MI, USA

**Keywords:** infant formula, human milk oligosaccharides, 2′-fucosyllactose, gastrointestinal tolerance, partially hydrolyzed whey

## Abstract

Human milk oligosaccharides are important components of breast milk. We evaluated feeding tolerance of the human milk oligosaccharide 2′-fucosyllactose (2′FL) in a 100% whey, partially hydrolyzed infant formula with the probiotic *Bifidobacterium animalis* ssp *lactis* strain Bb12 (*B lactis*; Test) as compared with the same formula without 2′FL (Control) in a randomized controlled trial of healthy infants enrolled at 2 weeks of age (±5 days). After 6 weeks of feeding the assigned formula, the primary outcome of tolerance was assessed using the Infant Gastrointestinal Symptom Questionnaire. Stooling, vomiting, spit-up, crying, and fussing were compared between groups. Seventy-nine infants were enrolled and 63 completed the study per protocol (30 Test, 33 Control). Infant Gastrointestinal Symptom Questionnaire scores were similar between groups (Test 20.9 ± 4.8, Control 20.7 ± 4.3, *P* = .82). Partially hydrolyzed infant formula with 2′FL and *B lactis* is tolerated well, as confirmed by a validated multi-symptom index.

## Introduction

Human milk oligosaccharides (HMOs) are unique and functional constituents of breast milk, being its third most abundant solid component, only lower in concentration than lactose and lipids.^1-3^ Approximately 200 different HMOs have been identified in breast milk with variable diversity and quantity of linear, branched, fucosylated, and sialylated HMOs occurring in all lactating women.^4^ 2′-Fucosyllactose (2′FL) is a trisaccharide composed of glucose, galactose, and fucose, and it is one of the HMOs occurring in greatest predominance in human milk.^3^ Levels of 2′FL vary in human milk depending on a woman’s secretor blood group status, ethnicity, and stage of lactation.^5^ Based on an international assessment of HMO levels in human milk at approximately 2 months postpartum among healthy women living in 8 countries, levels of 2′FL ranged from a mean of 0.7 ± 0.1 g/L in milk collected in Ghana to a mean of 3.4 ± 0.4 g/L in milk collected in California.^6^ In contrast to human milk, bovine milk contains low levels of oligosaccharides with low prevalence of fucosylated oligosaccharides.^7^

Evidence suggests that HMOs are important components of the innate immunity provided by human milk that help provide direct protection as well as contribute to the development of the infant’s immune system.^8^ 2′FL, in particular, serves as a selective prebiotic, an antiadhesive molecule against pathogens, and appears to modulate cellular immune responses.^2,7-9^ In vitro, 2′FL has been shown to block adhesion of *Campylobacter jejuni, Rotavirus, Norovirus*, and enterotoxigenic *Escherichia coli*, organisms known, in vivo, to cause infectious diarrhea in infants.^10-13^ Additionally, 2′FL has been shown to have direct cellular effects including ability to suppress inflammation, inhibit proliferation of peripheral blood mononuclear cells, and bind to lectins expressed on dendritic cells in vitro.^14-16^ Evidence from observational studies have further reflected a role of 2′FL in infant health. Infants breastfed by secretor mothers were reported to have enhanced levels of bifidobacteria and have earlier establishment of bifidobacteria-rich microbiota compared with infants breastfed by nonsecretor mothers.^17^ In an observational study of 93 breastfeeding mother-infant pairs, incidence of pathogen-specific diarrhea was significantly lower in infants who received 2′FL from their mothers’ milk compared with infants of nonsecretor mothers.^18^ In another study, Sprenger et al found significant associations between levels of 2′FL in mother’s breast milk and “any allergic disease,” immunoglobulin (Ig) E–associated disease, eczema, and IgE-associated eczema in Caesarean-section-born infants, with higher 2′FL levels associated with lower risk.^19^

Randomized clinical trials evaluating the effects of 2′FL added to infant formulas have demonstrated safety as well as potential clinical benefits. Initial evidence came from a trial evaluating effects of 2′FL and lacto-n-neotetraose in an intact whey and casein-based infant formula wherein normal growth and feeding tolerance were demonstrated; within the secondary outcomes, infants fed the formula with HMOs received fewer antibiotics and antipyretics than control-fed infants and had a gut microbiota closer to that of breastfed infants at the genus level.^20,21^ Another published trial similarly described appropriate growth in infants fed formulas containing 2′FL at levels of 0.2 and 1.0 g per liter of formula.^22^ Selective cytokine profiles of the infants fed formulas with 0.2 g/L or 1.0 g/L 2′FL were found to be similar to those of reference breastfed infants.^23^

Infant formulas with partially hydrolyzed whey protein (PHF-W) have beneficial effects on some manifestations of functional gastrointestinal (GI) disorders, such as regurgitation and stool consistency, and are often considered as nutrition options when formula-fed infants experience tolerance-related issues.^24,25^ Infant formula with a 100% whey, partially hydrolyzed protein base has also been shown to promote softer stools compared with stools of infants fed intact protein-based formula.^25^ The clinical trial described in this article was designed to assess the tolerance of this new PHF-W and probiotic *Bifidobacterium animalis* ssp *lactis* strain Bb12 (*B lactis*) with added 2′FL using a validated tool encompassing multiple parameters of feeding tolerance.^26^

## Methods

### Study Population

Healthy, full-term (≥37 weeks gestation; ≥2500 and ≤4500 g birth weight), singleton infants, ages 14 ± 5 days, who had been exclusively formula-fed for at least 3 days prior to enrollment were recruited for this trial. Infants already participating in a conflicting clinical trial and those being treated for reflux were excluded.

### Ethical Approval and Informed Consent

The study protocol was approved by the Chesapeake Research Review, Inc, Institutional Review Board, Columbia, Maryland, with protocol reference 00022536. Informed consent was obtained from the parents or guardians of participating infants. Good clinical practice was followed by all sites throughout the study. The study was registered with ClinicalTrials.gov (NCT# NCT03307122).

### Study Design

This was a randomized, controlled, double-blind multicenter study conducted from September 2017 to February 2018 at 7 sites throughout the United States. Subjects were enrolled at 14 ± 5 days of age after obtaining informed consent from their caregivers. Randomization to the Test or Control formula (Table 1) was performed by computer-generated assignment embedded in the centralized electronic data capture system. The randomization was performed in such a manner to assign subjects to formulas in a 1:1 ratio. Both formulas were made from 100% whey protein that was partially hydrolyzed, contained *B lactis*, and provided 0.67 kcal/mL and 2.2 g protein/L. The only difference between the 2 formulas was the addition of 0.25 g/L 2′FL to the Test formula. Subjects/caregivers, support staff, sponsor project manager, statisticians, and investigators were blinded as to the identity of the study formulas. The products were labeled with the same protocol number and color of label but were distinguished by the codes on the labels (4KN and MP9). At the enrollment visit (V0), the Infant Gastrointestinal Symptom Questionnaire (IGSQ) was administered and anthropometric measurements were taken by trained study staff. Caregivers began to feed the subjects with the randomly assigned formulas ad libitum after V0. After 42 days of feeding, subjects returned for a second visit (V1). For 2 days before V1, caregivers completed a diary of formula intake, stooling, spit-up, and vomit. At V1, the IGSQ and anthropometric measurements were repeated.

**Table 1.** Product Composition Declaration.

Manufacturer	Test	Control
Protein g/100 kcal	2.2	2.2
Protein source	100% whey partially hydrolyzed	100% whey partially hydrolyzed
Fat g/100 kcal	5.1	5.1
Fat source	Palm olein, soy, coconut, high-oleic safflower, ARA, DHA	Palm olein, soy, coconut, high-oleic safflower, ARA, DHA
Carbohydrates g/100 kcal	11.2	11.2
Carbohydrate source	Lactose, corn maltodextrin (70/30), 2′-fucosyllactose (0.25 g/L)	Lactose, corn maltodextrin (70/30)
*Bifidobacterium lactis*, CFU/g powder	1 × 10^6^	1 × 10^6^

Abbreviations: ARA, arachidonic acid; DHA, docosahexaenoic acid; CFU, colony-forming unit.

### The Infant Gastrointestinal Symptom Burden Questionnaire

An infant’s ability to tolerate a formula or food can be assessed based on several parameters, including stool patterns, frequency of spitting up, degrees of flatulence, and general demeanor or mood. Caregiver reports on these parameters can be combined to yield a GI symptom burden score using the IGSQ. The IGSQ is a validated 13-item questionnaire that assesses an infant’s GI-related signs and symptoms as observed by caregivers/parents over the previous week in 5 domains: stooling, spitting up/vomiting, flatulence, crying, and fussing.^25^ Caregivers/parents provide one response after each question is read to them by a trained interviewer, and items are scored on a scale of 1 to 5, with higher values indicating greater GI distress. Total IGSQ scores are calculated by summing item responses. The possible range in scores is 13 to 65, where a score of 13 indicates no GI distress and a score of 65 represents extreme GI distress. The IGSQ has been utilized successfully in clinical trials assessing infant feeding tolerance.^26-28^

### Two-Day Diary

Tolerance measures were documented by parents/caregivers over the 2 days prior to V1, which was the final visit, occurring after approximately 6 weeks of consumption of the Test and Control formulas. Caregivers were asked to record the amount of study formula consumed by the infant, the number of stools passed in a 24-hour period, consistency of each stool (watery, loose, soft, formed, hard), whether the infant had difficulty passing the stool (Yes or No), frequency of vomiting, spitting up (none, occasional [1-5 times/day], frequent [>5 times/day]), and durations of crying and fussing (<10, 10-30, 31-60, 61-120, 121-180, or more than 180 minutes). Spitting up was defined for caregivers/parents as “effortless return of small amounts of formula after feeding, usually about a mouthful” and vomiting as “a forceful return of larger amounts of formula.”

### Adverse Events

Adverse events (AEs) were collected throughout the study and were assessed by the site investigator or designee for duration, intensity, frequency, and relationship to test product. AEs were classified into System, Organ, and Class categories. Within the infections and infestations System, Organ, and Class category, 5 AE clusters were identified: upper respiratory tract infection, viral upper respiratory tract infection, otitis media/pharyngitis, thrush, and “other.”

### Sample Size

The primary objective of this clinical trial was to compare IGSQ scores after infants were fed the formula (Test) containing 2′FL or the formula without 2′FL (Control). The sample size was estimated for the primary endpoint based on an assumption of noninferiority with a limit of 4 points higher on the IGSQ for the Test versus the Control group, assuming an α level of .05 and 80% power. Sample size calculations were based on data from previous trials with similar study designs and endpoints. Based on these parameters, the total sample size calculated was 28 subjects per formula group. Eighty infants were recruited in order to account for a dropout rate of approximately 30%.

### Statistical Methods

Demographics and birth characteristics were analyzed using descriptive statistics and compared between formula groups using *t* tests for continuous measures and χ^2^ tests for categorical measures. IGSQ scores at V0 and V1 were compared between groups using *t* tests. Mean stool frequency per day over the 2-day diary period was compared between groups using a repeated measures model to account for multiple stools per day per baby. Stool consistency in each category was examined using percentages based on all stools passed and compared between groups first using a repeated measures ordinal model with 5 stool consistency categories. Additionally, stool consistency was grouped into categories of Soft + Loose versus Watery + Formed + Hard and examined in a repeated measures model. Difficulty in passing stool was examined at the subject level with a between-group comparison using Fisher’s exact test due to small cell counts. Crying, fussing, and vomiting frequency were compared using a repeated measures model to account for multiple measurements over the 2-day diary period for each baby. Spitting up was assessed first as a Yes/No variable for each baby on each diary day and examined with a repeated measures model. Next, frequency of spit-up was evaluated at the level of the baby as either Yes (spit-up on one or both diary days) versus No (no spit-up on either diary day) with comparisons made between groups using a *t*-χ^2^ test. Weights and weight-for-age *z* scores and percentiles, as well as length and length-for-age *z* scores and percentiles, were calculated according to the World Health Organization growth charts (https://www.cdc.gov/nccdphp/dnpao/growthcharts/resources/sas-who.htm) and length with strata of gender at each study visit. AEs were counted within formula groups at the event level as well as at the subject level.

All statistical analyses were performed using Stata (StataCorp, 2017; Stata Statistical Software: Release 15; StataCorp LLC, College Station, TX) or SAS/STAT software, Version 9.4 of the SAS System for Windows (SAS Institute Inc, Cary, NC). Statistical significance was assessed using an α level of 5% with 2-sided statistical tests unless otherwise specified.

The Intent-To-Treat (ITT) population was defined as all randomized subjects who took any amount of the study formula, while the Per Protocol (PP) population was defined by excluding subjects if they were unlikely to have had full exposure to the study formulas as defined by these criteria: had a break in study formula feeding longer than 3 days, were hospitalized for more than 3 days, had more than 4 teaspoons of complementary foods per day, and/or had more than 3 ounces of juice per day. The primary outcome of IGSQ scores at V1 was analyzed in the ITT and PP populations. Secondary outcomes including safety endpoints were analyzed only in the ITT population.

Customized, secure electronic case report forms and database (Medidata Solutions, Inc, New York, NY) were used to collect and store data. Study staff entered data into the eCRF database, and data were quality checked by a data manager before database lock.

## Results

**Figure 1.**
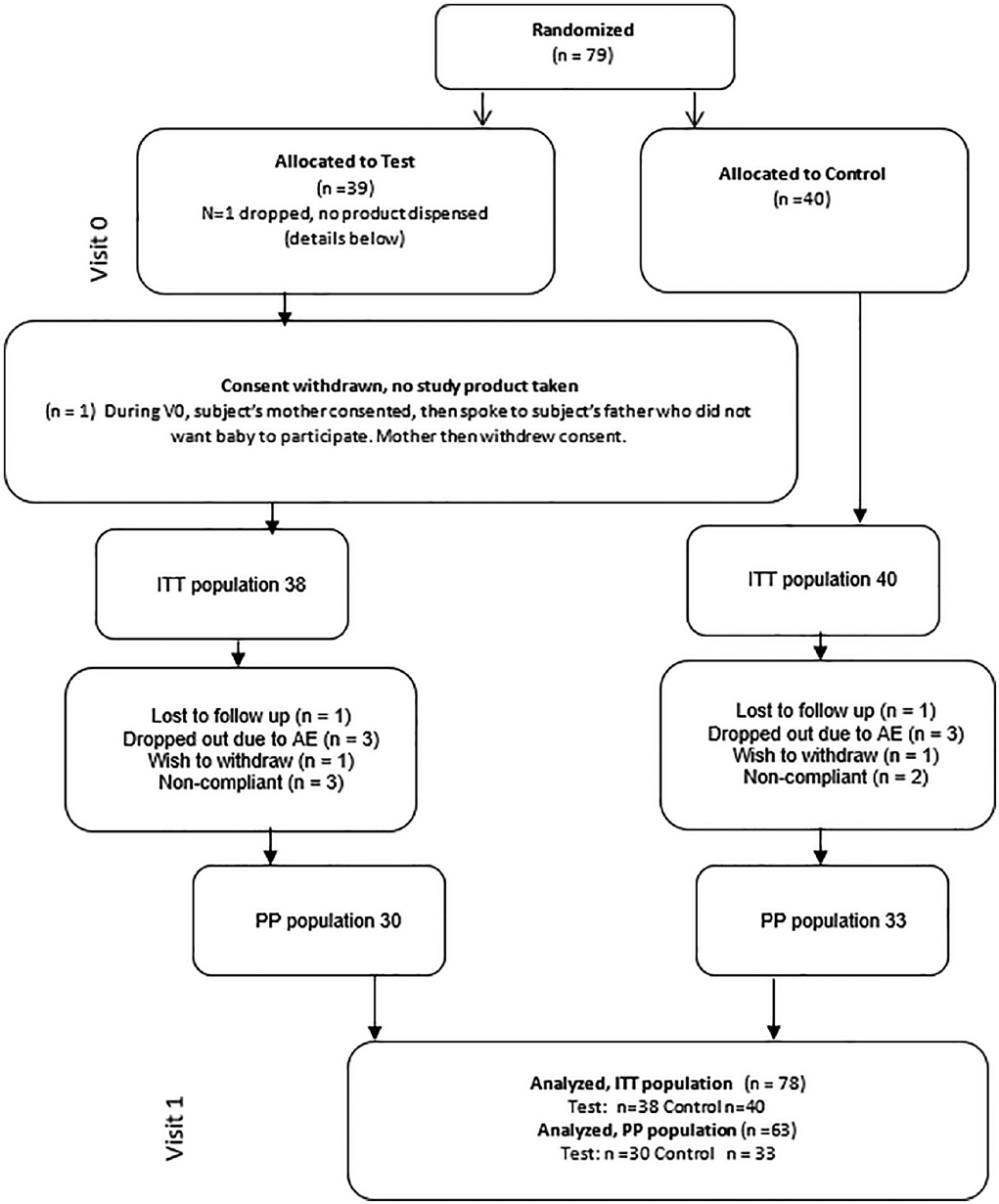
Flow diagram of subject randomization, allocation, and analytic populations.

Seventy-nine subjects were randomized. One subject withdrew consent before receiving study product. Seventy-eight subjects were, therefore, included in the ITT analyses (38 Test, 40 Control). In the Test group, 1 subject was lost to follow-up, 1 caregiver wished to withdraw, 3 withdrew due to AEs, and 3 were noncompliant with feeding only study formula. In the Control group, 1 subject was lost to follow-up, 1 caregiver wished to withdraw, 3 withdrew due to AEs, and 2 were noncompliant. Therefore, 30 subjects from the Test group and 33 subjects from the Control group were included in the PP analysis (Figure 1).

### Demographics

Overall, subjects were at a mean age of 14 (±3.3) days at time of enrollment. The demographics of the ITT population are shown in Table 2. There were no differences in gender, ethnicity, delivery type, gestational age at birth, birth anthropometrics, whether the subject had siblings, maternal age, or maternal education between groups in the ITT population. The Test group had significantly more infants who were reported as having ever been breastfed compared with the Control group (20 [53%] vs 9 [22%], *P* = .006). The mean number of days of breastfeeding prior to enrollment, however, were not significantly different between groups (Test: 4.7 [± 4.0] days vs Control 3.6 [±4.7] days, *P* = .51).

**Table 2.** Demographics, Intent-To-Treat Population.

	Test (N = 38), n (%) or Mean (SD)	Control (N = 40), n (%) or Mean (SD)	Total (N = 78), n (%) or Mean (SD)
Gender			
Male	20 (53%)	25 (63%)	45 (58%)
Female	18 (47%)	15 (37%)	33 (42%)
*P*	.38		
Ethnicity			
Asian	0 (0%)	0 (0%)	0 (0%)
Black	23 (60%)	19 (48%)	42 (54%)
Caucasian	14 (37%)	16 (40%)	30 (39%)
Hispanic	1 (3%)	4 (10%)	5 (6%)
Other	0	1 (2%)	1 (1%)
*P*	.38		
Delivery type			
Vaginal	24 (63%)	23 (58%)	47 (60%)
Cesarean	14 (37%)	17 (42%)	31 (40%)
*P*	.61		
Age at enrollment, days (mean [SD])	14.1 (3.1)	13.9 (3.5)	14.0 (3.3)
*P*	.76		
Gestational age at birth, weeks (mean [SD])	39.0 (1.1)	38.9 (1.1)	38.9 (1.1)
*P*	.47		
Weight at birth, g (mean [SD])	3334 (468)	3307 (396)	3320 (430)
*P*	.78		
Length at birth, cm (mean [SD])	50.3 (2.6)	50.4 (2.2)	50.4 (2.4)
*P*	.92		
Siblings			
No	10 (26%)	14 (35%)	24 (31%)
Yes	28 (74%)	26 (65%)	54 (69%)
*P*	.41		
Mother’s age	27.9 (5.9)	26.9 (6.3)	27.4 (6.1)
*P*	.48		
Highest level of maternal education			
Grade school	1 (3%)	1 (2%)	2 (2%)
High school	16 (42%)	18 (45%)	34 (44%)
Some college	13 (34%)	12 (30%)	25 (32%)
College	6 (16%)	7 (18%)	13 (17%)
Other	2 (5%)	2 (5%)	4 (5%)
*P*	.99		
Baby ever breastfed			
No	18 (47%)	31 (78%)	49 (63%)
Yes	20 (53%)	9 (22%)	29 (37%)
*P*	.006		
Mean (SD), days of breastfeeding	4.7 (4.0)	3.6 (4.7)	4.3 (4.2)
*P*	.51		

### Primary Outcome: Infant Gastrointestinal Symptom Questionnaire

The primary outcome was comparison of IGSQ scores for the PP population between groups at V1, occurring after 6 weeks of feeding. IGSQ scores for the Test and Control group were similar at baseline for both ITT and PP analyses (ITT: 21.0 ± 7.1 vs 21.7 ± 8.2 in Test vs Control, *P* = .68, and PP: 20.1 ± 6.6 vs 20.7 ± 7.5 in Test vs Control, *P* = .77). At V1, mean IGSQ scores were similar between groups (ITT: 22.5 ± 6.4 vs 20.8 ± 4.5 in Test vs Control, *P* = .19, and PP: 20.9 ± 4.8 vs 20.7 ± 4.3 in Test vs Control, *P* = .82) and within the hypothesized 4-point limit of noninferiority for both ITT and PP analyses (Table 3).

**Table 3.** Infant Gastrointestinal Symptom Questionnaire (IGSQ) Scores, PP and ITT Populations.

Visit 0, Enrollment	ITT		PP	
	Test (N = 38)	Control (N = 40)	Test (N = 30)	Control (N = 33)
	Mean (SD), Median (Min, Max)	Mean (SD), Median, (Min, Max)	Mean (SD), Median, (Min, Max)	Mean (SD), Median, (Min, Max)
Total IGSQ score	21.0 (7.1), 20.5, (13.0, 46.0)	21.7 (8.2), 19.0, (13.0, 50.0)	20.1 (6.6), 19.5, (13.0, 46.0)	20.7 (7.5), 18.0, (13.0, 50.0)
*P* ^a^	.68		.77	
Visit 1, After 6 Weeks	Test (N = 35)	Control (N = 35)	Test (N = 30)	Control (N = 33)
	Mean (SD), Median, (Min, Max)	Mean (SD), Median, (Min, Max)	Mean (SD), Median, (Min, Max)	Mean (SD), Median, (Min, Max)
Total IGSQ score	22.5 (6.4), 22.0, (14.0, 34.0)	20.8 (4.5), 21.0, (13.0, 30.0)	20.9 (4.8), 20.0, (14.0, 34.0)	20.7 (4.3), 21.0, (13.0, 30.0)
*P* ^a^	.19		.82	

Abbreviations: PP, Per Protocol; ITT, Intent-To-Treat; min, minimum; max, maximum.

a *P* value from *t* test.

### Secondary Outcomes

#### Stool Frequency, Consistency, and Ease of Passing

**Figure 2.**
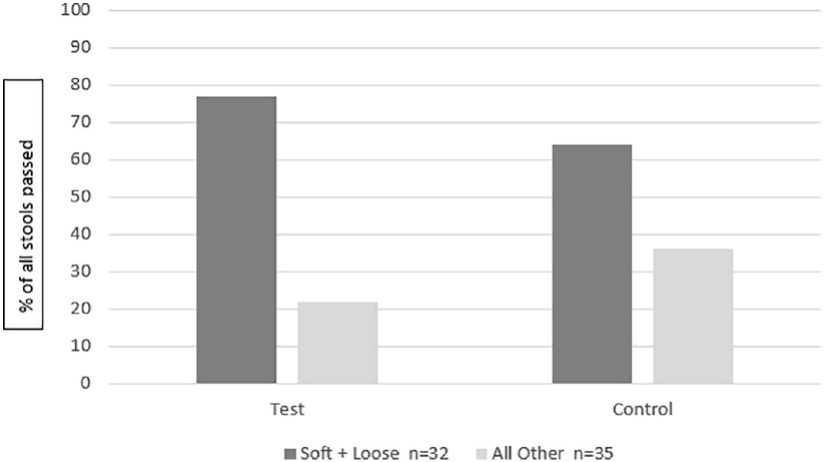
Mean caregiver reported stool consistency percentages over 2-day diaries at Visit 1, intent-to-treat population.

Based on data recorded by caregivers in the 2-day diaries, stool frequency did not differ between groups; infants in the Test group passed 2.2 (±1.8) stools per day and infants in the Control group passed 2.3 (±2.1) stools per day in the 2-day period prior to the final visit (*P* = .79). Stool consistency, based on an ordinal model utilizing 5 stool consistency categories, did not differ significantly between groups (*P* = .65) in the ITT population. Stools of infants fed the Test formula were either loose or soft 77% of the time (Figure 2).

More stools were reported to be difficult to pass in the Control group (4 [3%] Test and 33 [21%] Control, *P* = .04); however, the number of infants with stools reported as difficult to pass stools did not differ (4 [13%] in Test vs 10 [29%] in Control, *P* = .14).

#### Spit Up, Vomiting, Crying, and Fussing

Crying and fussing duration and vomiting frequency were similar between groups in the ITT and PP populations. The proportion of babies reported to have any spit-up over the 2-day diary period did not differ between groups (79% Test, 64% Control, *P* = .18); however, among the babies whose caregivers reported spit-up, significantly more were reported to have spit-up >5 times per day in the Test group than the Control group.

#### Formula Intake

Average formula consumption volumes did not differ between formula groups. Infants in the Test formula group consumed 24.8 (±13.7) ounces per day and infants in the Control formula group consumed 24.1 (±12.0) ounces per day.

#### Adverse Events

There were no serious AEs reported in the study. Seventy-two AEs occurred in the study, 36 in the Test group and 36 in the Control group, corresponding to 17 and 19 subjects in the Test and Control groups, respectively. Spit-up reported as an adverse event was of interest due to the finding that there were more subjects with spit-up noted as “frequent” in the Test group compared with the Control group; however, only one subject in each group reported “mild” spit-up as an AE, and no subjects had reports of more extreme spitting up.

In the category of reported infections and infestations, there were more subjects with this category of AE in the Control versus the Test Group (Control 9 [23%] vs Test 3 [8%], *P* = .05). A *P* value of .05 is marginally significant and suggestive of a possible association between 2′FL and the lower rate of infections. However, the small number of cases experiencing infections suggests interpreting this *P* value with caution. Looking specifically at upper respiratory infections, there was a higher but nonsignificant incidence in the Control 4 (10%), versus Test 0 (0%), *P* = .12. Overall, there were no safety concerns noted with either of the study formulas.

#### Anthropometrics

Body weights and lengths were obtained at Visits 0 and 1. Weights and lengths were similar between groups at V0 and at V1, after 6 weeks on the study formulas for both boys and girls. Weight-for-age and length-for-age percentiles for both study visits are shown in Table 4.

**Table 4.** Anthropometry at 2 and 8 Weeks of Age, Intent-To-Treat Population, World Health Organization Growth Charts.

	Test			Control		
Visit 0, Enrollment	Boys, N = 20	Girls, N = 18	Combined, N = 38	Boys, N = 25	Girls, N = 15	Combined, N = 40
Weight-for-age percentile	51.1 ± 27.0	35.2 ± 23.2	43.5 ± 26.2	40.6 ± 20.8	47.7 ± 21.3	43.3 ± 21.0
Length-for-age percentile	51.0 ± 31.2	37.6 ± 24.1	44.7 ± 28.5	40.6 ± 31.1	52.4 ± 27.0	45.0 ± 29.8
Visit 1, After 6 Weeks	Boys, N = 18	Girls, N = 17	Combined, N = 35	Boys, N = 23	Girls, N = 12	Combined, N = 35
Weight-for-age percentile	49.5 ± 25.4	42.1 ± 27.4	45.9 ± 26.3	39.6 ± 25.2	48.9 ± 25.8	42.8 ± 25.4
Length-for-age percentile	53.6 ± 38.0	54.2 ± 30.0	53.9 ± 33.9	50.4 ± 33.0	59.2 ± 34.3	53.4 ± 33.2

## Discussion

This prospective randomized controlled trial demonstrates with a validated GI burden index that 2′FL added to partially hydrolyzed whey formula with a probiotic is well tolerated. This finding is similar to conclusions made from other works evaluating feeding tolerance without use of a validated tools like the IGSQ tool in infants fed formulas containing 2′FL combined with lacto-n-neotetraose and 2′FL combined with galacto-oligosaccharides.^20,22^ Tolerance is an important outcome both for health care practitioners and caregivers of infants, with estimates of over one third to nearly half of infants having their formula switched at least one time, usually for parent-perceived intolerance.^29,30^ Objective assessments of a formula’s tolerance provide a basis on which pediatric health professionals can make decisions. The IGSQ cohesively evaluates 5 separate domains of feeding tolerance, assigning each a degree of severity and arriving at a symptom burden score, making the results of this clinical trial relevant for the practicing clinician.

Data from the 2-day diaries, completed by caregivers, provided further demonstration that the Test formula was tolerated. The stool consistency findings correspond to previous clinical trial data from infants fed formula based on partially hydrolyzed whey, where the proportion of stools reported as soft was predominant on a 4-point scale of watery, soft, formed, and hard.^25,31^ Partially hydrolyzed whey formulas are devoid of intact bovine proteins and are frequently initiated when infants are perceived to have intolerance of formulas containing intact casein and whey. The mechanisms supporting the tolerance of PHF-W are not well defined; however, their faster rate of gastric emptying and production of softer stools versus intact whey and casein-based formulas may be influential.^25,32^ Incidence of crying, fussiness, vomiting, and spit-up based on data from the 2-day diaries were similar between groups with the exception that more subjects on Test formula had reports of “frequent” spit-up. Spitting up was not an issue for either product overall, as only one subject in each group had spit-up reported as an AE. More importantly, in both of these subjects, the relationships to the study product were deemed by the site investigators to be “unlikely” or “unrelated” to study formula. Spit-up frequency was not found to be influenced by 2′FL in other infant formula trials evaluating levels of 2′FL similar to and higher than the level in the Test formula in this study.^20,22^ Consistent with prior studies, the Test formula of this study with 2′FL was well tolerated.

The breastfed infant has an intestinal microbiome unique from that of the formula-fed infant, as influenced in part by the presence of bacteria and HMOs in mother’s milk. Probiotics and oligosaccharides have been introduced to infant formula to help shift feeding outcomes closer to those of the human milk–fed infant. Both Test and Control formulas evaluated in this trial contained the probiotic *B lactis*. While previous work has demonstrated no effect of *B lactis* on feeding tolerance, *B lactis* in infant formula has been associated with its increased presence in stool and increased concentrations of fecal secretory–specific, anti-rotavirus–specific, and anti-poliovirus–specific IgA.^31^ Combinations of HMOs and probiotics offer the potential to bring more of the benefits of the breastmilk to the formula-fed infant; yet, more research is needed to evaluate the effects of diverse combinations on the microbial signature and specific health outcomes in infants.

The tendency toward fewer infections in the Test group versus the Control group suggests a possible effect of 2′FL on supporting the developing immune function in infants. Although based on a small number of subjects, the finding is directionally similar to significant reductions in infections observed in breastfed infants receiving human milk found to be rich in 2′FL and calls for further study in a larger population.

This study has a few key strengths. The only difference between the Test and Control formulas was the inclusion of 2′FL in the Test formula, allowing for differences in outcomes to be associated with 2′FL alone. This is in contrast to other trials evaluating HMOs in infant formula, where combinations of oligosaccharides were studied. Another strength was the use of the validated IGSQ that evaluated 5 separate domains of GI burden and combined them to arrive at single cohesive scores. This primary outcome was combined with tolerance data collected via a 2-day diary that allowed for a more thorough assessment of the frequency and severity of GI symptoms associated with feeding the study formulas. Additional strengths of this tolerance assessment included the enrollment of infants through multiple centers across the country. The only difference between formula groups at baseline was that there were more infants in the Test group versus the Control group that had ever received breast milk (53% vs 22%). Given that the mean duration of breastfeeding was short (Test 4.7 ± 4.0 days, Control 3.6 ± 4.7 days) and the duration of study feeding was 6 weeks, the difference in breastfed days is considered unlikely to influence tolerance outcomes. One limitation of this study was that the tolerance assessment was limited to a level of 2′FL at the lower range of what has been observed in human milk. A higher level of 2′FL could provide more insight into the effects of this HMO on GI tolerance.

In conclusion, an infant formula with 100% whey, partially hydrolyzed, as the protein source with the addition of 0.25 g/L of the HMO 2′FL and probiotic *B lactis* is tolerated well based on a comprehensive tolerance assessment tool and is tolerated similarly to an otherwise identical formula without 2′FL.
